# GPR171 activation regulates morphine tolerance but not withdrawal in a test-dependent manner in mice

**DOI:** 10.1097/FBP.0000000000000692

**Published:** 2022-08-05

**Authors:** Leela Afrose, Max V. McDermott, Ashif I. Bhuiyan, Sanjai K. Pathak, Erin N. Bobeck

**Affiliations:** a Department of Biology; b Interdisciplinary Neuroscience Program, Utah State University, Logan, Utah; c Chemistry and Biochemistry Department, Queens College of The City University of New York, Flushing, New York; d Chemistry Doctoral Program; e Biochemistry Doctoral Program, The Graduate Center of The City University of New York, New York

**Keywords:** GPCR, GPR171, GPR171 agonist, morphine tolerance, sex differences, withdrawal

## Abstract

A newly deorphanized G protein-coupled receptor, GPR171, is found to be highly expressed within the periaqueductal gray, a pain-modulating region in the brain. Our recent research has shown that a GPR171 agonist increases morphine antinociception in male mice and opioid signaling *in vitro*. The objective of this study was to evaluate the effects of combination treatment in females as well as whether chronic treatment can be used without exacerbating morphine-induced tolerance and withdrawal in female and male mice. Our results demonstrate that activation of GPR171 with an agonist attenuates morphine tolerance in both female and male mice on the tail-flick test, but not the hotplate test. Importantly, the GPR171 agonist in combination with morphine does not exacerbate morphine-induced tolerance and withdrawal during long-term morphine treatment. Taken together, these data suggest that the GPR171 agonist may be combined with morphine to maintain antinociception while reducing the dose of morphine and therefore reducing side effects and abuse liability. The outcome of this study is clearly an important step toward understanding the functional interactions between opioid receptors and GPR171 and developing safer therapeutics for long-term pain management.

## Introduction

The yearly cost of chronic pain is approximately $635 billion in the United States, which is greater than the combined annual costs of heart disease, cancer and diabetes (Gaskin and Richard, 2012). Management of chronic pain is considered as one of the pivotal issues in public healthcare (Cherubino *et al.*, 2012). Opioid analgesics, such as morphine, represent the gold standard pain killer frequently used for the treatment of moderate to severe pain. Despite being a potent analgesic, the utility of morphine for the treatment of chronic pain is restricted due to the development of tolerance and withdrawal. Better pain therapeutics are needed that produce less severe side effects.

G protein-coupled receptors (GPCRs) are an attractive therapeutic target due to their high cell surface expression, their role in initiating cell signaling and their ability to modulate many pathophysiological processes, including pain. Previous studies have shown that almost 35% of all Federal Drug Administration-approved drugs act by targeting GPCRs (Insel *et al.*, 2019) and there are around 40 members of the GPCR superfamily that have the potential to modulate pain (Stone and Molliver, 2009). Recently our lab found that a newly deorphanized GPCR, GPR171, can modulate morphine antinociception and is highly expressed within areas involved in pain modulation including the periaqueductal gray (PAG) and the spinal cord (McDermott *et al.*, 2019). The PAG is also a key brain region involved in opioid antinociception, tolerance and withdrawal (Behbehani, 1995; Hao *et al.*, 2011; Bobeck *et al.*, 2012). Given that we recently found that GPR171 ligands modulate opioid signaling and antinociception in male mice (McDermott *et al.*, 2019), combination therapy with morphine and a GPR171 agonist may be a useful pain therapeutic. However, the side effect profile of this GPR171 agonist must be carefully investigated before significant resources are invested in drug discovery efforts. In addition, studies evaluating GPR171 in females are needed given that to date only one article has used females, which found reduced effects in chronic pain compared to males (Ram *et al.*, 2021). The purpose of this study was to evaluate whether repeated treatment of morphine in combination with a GPR171 agonist leads to greater side effects, such as tolerance and withdrawal in male and female mice.

## Methods

### Subjects

Male and female C57BL/6 mice (*n* = 158) (Charles River Laboratories, California, USA) were used. Mice were 6–13 weeks old and weighed 13-27 g at the beginning of the experiment. Animals were housed (4–5 per cage) in a humidity and temperature-controlled room with a 12:12 hour light/dark (lights on at 07:00) cycle. Mice were habituated in the testing room and handled for 3 days before testing. All procedures were conducted in compliance with the guidelines by the International Association for the Study of Pain approved by the Utah State University Institutional Care and Use Committee (Protocol #2775). A within-subjects design and a cumulative dosing procedure were utilized to reduce the number of animals in these experiments.

### Synthesis of GPR171 agonist, sodium 5-methacrylamidoisophthalate (MS15203)

Methacrylic acid (4.3 mmol, 0.37 g) was dissolved in anhydrous N, *N*-dimethylformamide (10 ml) and activated by the addition of an amide coupling reagent, O-(1H-6-chlorobenzotriazole-1-yl)-1,1,3,3-tetramethyluronium hexafluorophosphate (4.3 mmol, 1.8 gm). To this was added dimethyl-5-aminoisopthalate (1.4 mmol, 0.29 g) and N, *N*-diisopropylethylamine (12.9 mmol, 1.67 gm) dropwise. The resulting mixture was stirred for 30 min in the ice bath under an inert nitrogen environment. The reaction mixture was then allowed to stir overnight at room temperature. After the reaction was complete, as monitored with thin-layer chromatography, the reaction mixture was diluted with dichloromethane (15 ml) and was transferred to a separatory funnel. The organic layer was washed with deionized water (10 ml, 2×) and brine (10 mL). The organic layer was dried with anhydrous sodium sulfate and the solvent was removed with the rotary evaporator. The product dimethyl 5-methacrylamidoisophthalate was dried overnight under high vacuum and its presence was confirmed by ^1^H NMR. It was utilized in the next and final step of synthesis without further purification. Dimethyl 5-methacrylamidoisophthalate (1.43 mmol, 0.4 g), dissolved in tetrahydrofuran (10 mL), was cooled on ice bath. To this was added sodium hydroxide [7.15 mmol (0.29 g) in 5 mL water] and the resulting mixture was stirred vigorously overnight. After the reaction was complete, it was diluted with water (15 mL) and washed with ethyl acetate (10 mL, 2×). The aqueous layer was acidified with a concentrated hydrochloric acid solution to a pH of 1.5 and washed with ethyl acetate (10 mL, 2×). The collected organic layer was dried on anhydrous sodium sulfate_,_ solvent removed with the rotary evaporation and the product 5-methacrylamidoisophthalic acid dried overnight under high vacuum. To obtain the sodium 5-methacrylamidoisophthalate, a more water-soluble form of the compound MS15203, 5-methacrylamidoisophthalic acid was treated with saturated sodium bicarbonate solution and subjected to final purification by C-18 reverse phase HPLC using acetonitrile/water gradient. ^1^H NMR (400 MHz, DMSO-d6; TMS); δ ppm: 13.31 (s, 2H), 9.32 (s, 1H), 8.46 (s, 2H), 8.16 (s, 1H), 5.75 (s, 2H), 2.06 (s, 3H); ^13^C NMR (400 MHz, DMSO-d6; TMS); δ ppm: 170.18, 170.18, 166.26, 157.66, 131.77, 131.77, 124.72, 124.72, 124.72, 123.44, 20.66; Mass spectrometry (elctrospray ionization) calculated for C_12_H_9_NNa_2_O_5_, theoretical: 293.03, found: 293.08.

### Drug treatments

**Fig. 1:**
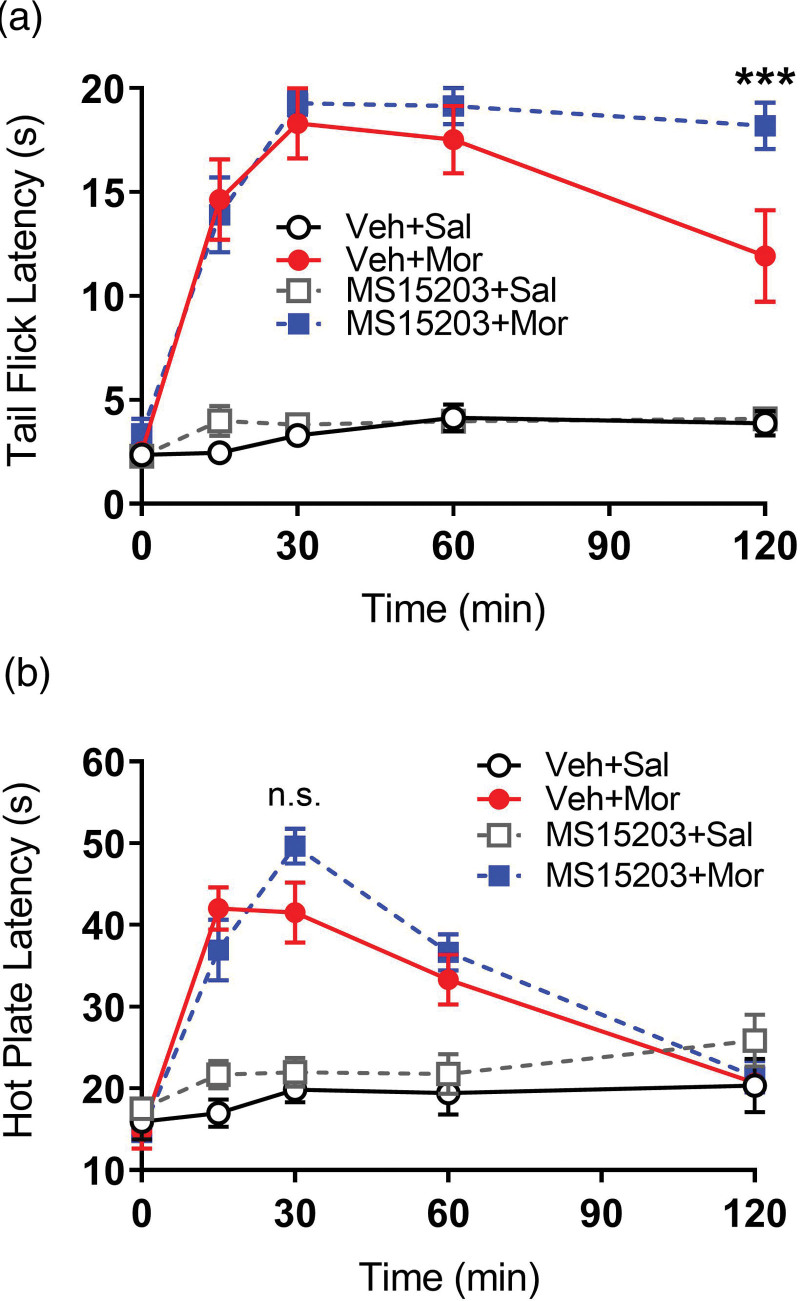
Effects of GPR171 agonist, MS15203, on acute morphine antinociception on the tail-flick and hot plate tests in female mice. Animals were injected with MS15203 (10 mg/kg, i.p.) or vehicle (10% DMSO, i.p.) followed 10 min later by an injection of morphine (5 mg/kg, s.c.) or saline (10 ml/kg, s.c.). Antinociception was assessed at 15, 30, 60 and 120 min time points after the second injection using the (a) tail flick warm water test (52 °C) and (b) hot plate test (50 °C). Administration of MS15203 enhanced morphine-induced antinociception at 120 min time point on the tail flick test but not on the hot plate test. ***P < 0.001, *n* = 7–9 animals/group. DMSO, dimethyl sulfoxide.

GPR171 agonist, MS15203 (sodium 5-methacrylamidoisophthalate, 10 mg/kg; synthesized as described above) was dissolved in 10% dimethyl sulfoxide (DMSO) in 0.9% saline (ChemBridge Co., San Diego, California, USA) or 0.9% saline. In our initial study MS15203 (ChemBridge) caused an enhancement of morphine antinociception in male mice (McDermott *et al.*, 2019) which is similar to what was found in female mice in the current study using the synthesized water-soluble MS15203 (see Fig. 1). The synthesized MS15203 was used in all experiments except in the tolerance experiment in males. Morphine Sulfate (5 mg/kg, Hikma, Eatontown, New Jersey, USA) was dissolved in 0.9% saline. All drugs were administered at a volume of 10 mL/kg. These doses were chosen based on previous studies (Wardman *et al.*, 2016; Bobeck *et al.*, 2017; McDermott *et al.*, 2019). Male and female mice were randomly divided into four groups: Vehicle+Morphine, Vehicle+Saline, MS15203+Morphine and MS15203+Saline. To perform a dose-response curve, a within-subjects design was used where cumulative quarter-log doses (1, 1.8, 3.2, 5.6, 10 and 18 mg/kg) of morphine were administered to all mice. Naloxone hydrochloride (2 mg/kg, Tocris Bioscience) dissolved in 0.9% saline was used to precipitate morphine withdrawal.

### Behavioral assessment

Thermal nociception was assessed using the hot plate (Harvard Apparatus, Holliston, Massachusetts. USA) and warm water tail flick (Thermo Fisher Scientific) tests. On the hot plate test, mice were placed on a 50 °C hot plate and the latency to lick the hind paw was measured. The hot plate test involves both cerebral and spinal mediated circuits and is considered to be a supra-spinally organized response. Mice were removed from the hot plate if no response occurred within 60 s to avoid tissue damage.

The tail-flick test was conducted using a 52 °C warm water bath. The tip (5 cm) of the tail was immersed in warm water and their tail-withdrawal latency (rapid flick) was measured. The tail flick test is considered to be a spinal reflex response. In this test, the cutoff time was 20 s. No additional injections or testing were performed once the mice reached this cutoff latency.

### Data and statistical analyses

Statistical analyses of data were conducted by using Microsoft Excel and Prism 8.0 software (GraphPad). Data were analyzed by one-way or two-way ANOVA (repeated measures) when appropriate using Prism software. Dunnett’s or Tukey’s honestly significant difference post hoc tests were conducted to make pairwise comparisons. Raw data for the dose-response curves were converted to % maximum possible effect using the following equation: (latency − baseline latency)/(cutoff latency − baseline latency) × 100 to control for variations in baseline scores. Half maximal effect (ED50 ± SE) was calculated using nonlinear regression with a variable slope as described previously (Bobeck *et al.*, 2014). The bottom of the curve is defined as the average baseline score and the top as the cutoff score for the respective behavioral test (20s = tail flick and 60s = hotplate). Antinociceptive tolerance is defined as a statistically significant increase in the dose that produces 50% antinociceptive effect (ED50). ED50 values were analyzed using a one-way ANOVA for each sex independently. This approach to evaluate drug tolerance has been used numerous times previously by our lab and others (Tallarida, 2000; Morgan *et al.*, 2006; Eidson and Murphy, 2013; Bobeck et al., 2014, 2019; Eidson *et al.*, 2017). Statistical significance was defined as a probability less than 0.05.

### Experiment 1: antinociceptive time course in female mice

Following baseline measurements, mice were injected with MS15203 (10 mg/kg, i.p.) or vehicle 10% DMSO (10 mL/kg, i.p.). Ten minutes later, mice were administered a subcutaneous injection of morphine (5 mg/kg) or an equal volume of saline (10 mL/kg). After this second injection, mice were tested on the hot plate and tail flick tests at 15, 30, 60 and 120 min.

### Experiment 2: tolerance induction and dose-response paradigm

On day 1, mice were injected with MS15203 (10 mg/kg, i.p.) or 10% DMSO (10 mL/kg, i.p.) followed by a subcutaneous injection of morphine (5 mg/kg) or saline (10 mL/kg) 10 min later as in Experiment 1. On days  1–4, mice were injected with their designated drug combination twice daily, once in the morning and once in the afternoon (at least 6 h apart, approximately 10:00 and 16:00) to induce morphine tolerance as done previously. On the morning of day 5, morphine was administered to all animals regardless of pretreatment group designation using a cumulative dosing procedure to obtain final doses of 1, 1.8, 3.2, 5.6, 10 and 18 mg/kg. Injections were given 30 min apart and hot plate and tail flick latencies were assessed 15 min following each injection. These doses and injection times were adopted and modified from previous studies in rats and in mice during our preliminary studies (Ingram *et al.*, 2007; Bobeck *et al*., 2009, 2014).

### Experiment 3: morphine dependence and withdrawal paradigm

Mice were treated on days 1–4 as in experiment 2. On day 5, each mouse received their designated drug combination in the morning only. Two hours later an intraperitoneal injection of naloxone hydrochloride (2 mg/kg) was given. Immediately after the naloxone injection, mice were placed into a plexiglass cage, monitored and video recorded for 30 min to evaluate their withdrawal behaviors. Given that jumping is considered a well-documented withdrawal behavior in mice (Kest *et al.*, 2002), number of jumps were observed and counted as withdrawal behavior by a blinded observer.

## Results

### GPR171 agonist alters acute morphine antinociception in female mice

To assess the role of the GPR171 agonist, MS15203, on acute morphine antinociception in female mice, tail flick and hot plate thermal pain assays were run in the same animals with the hot plate first (Fig. 1). As expected, a repeated measures two-way ANOVA revealed a statistically significant difference in tail flick latencies between drug treatments [main effect drug: *F* (3, 28) = 104.2; *P* < 0.001] across time [main effect time: *F* (4, 112) = 47.34; *P* < 0.001], as well as a significant interaction (drug X time) [*F* (12, 112) = 12.01, *P* < 0.001] (Fig. 1a). Before drug administration, there was no statistically significant difference in the baseline tail flick latencies between any group (Tukey’s, NS). Vehicle+Morphine and MS15203+Morphine produced greater tail flick latencies at 15, 30, 60 and 120 min compared to ehicle+Saline (Tukey’s *P* < 0.05). At the 120 min time point, the MS15203+Morphine treatment produced significantly greater antinociception than Vehicle+Morphine (Tukey’s *P* < 0.05). However, at the 15, 30 and 60 min time points both morphine groups produced near maximal cutoff latency and there was no statistically significant difference between the MS15203+Morphine group and Vehicle+Morphine group (Tukey’s, NS) indicating that pretreatment with MS15203 only enhanced morphine antinociception on the tail flick test at the last time point (Fig. 1a).

Similarly, a repeated measures two-way ANOVA revealed an overall statistically significant difference in hot plate latencies between drug treatments [main effect drug: *F* (3, 28) = 20.48, *P* < 0.001], across time points [main effect time: *F* (4, 112) = 40.80, *P* < 0.001], and interaction (drug X time) [*F* (12, 112) = 12.39, *P* < 0.05] (Fig. 1b). Baseline hot plate latencies were not statistically different between any group before starting the experiment (Tukey’s, NS). Vehicle+Morphine and MS15203+Morphine produced greater hot plate latencies compared to Vehicle+Saline treated group at 15, 30 and 60 min (Tukey’s *P* < 0.05). While the MS15203+Morphine group showed a slight increase in antinociception, it was not statistically significant (Tukey’s, NS)) compared to the Vehicle+Morphine group at any timepoint (Fig. 1b). Overall, these data suggest that GPR171 agonist, MS15203, enhanced acute morphine antinociception at one time point on the tail-flick test, but not on the hot plate test in female mice (Fig. 1).

### GPR171 agonist reduces morphine tolerance in female mice on the tail flick test

**Fig. 2:**
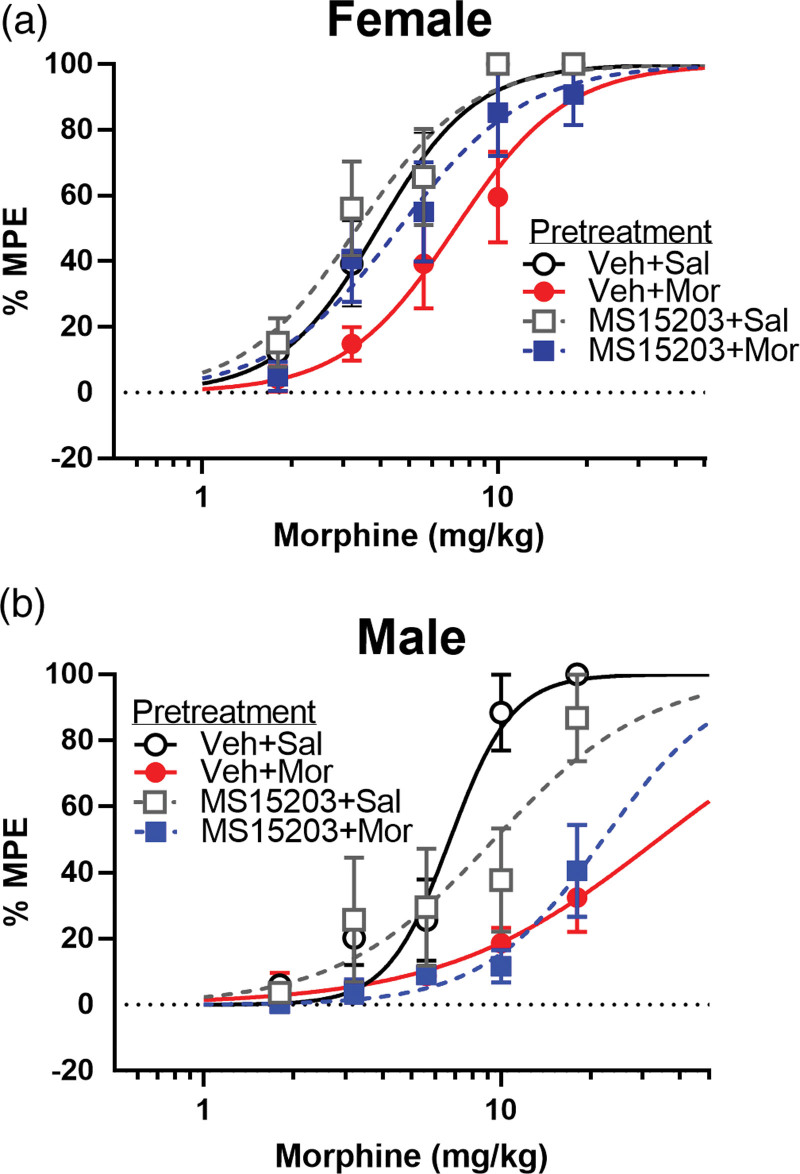
GPR171 agonist, MS15203, reduced morphine tolerance in female and male mice on the tail-flick test. Morphine tolerance was induced by twice daily injections of morphine for 4 consecutive days. On day 5, cumulative quarter-log doses of morphine (1, 1.8, 3.2, 5.6,10 and 18 mg/kg) were injected subcutaneously to perform a dose-response curve. Tolerance was evident by the rightward shift in the dose-response curve in the morphine treated group compared to saline treated mice. (a) Repeated administration of MS15203+Morphine was not significantly different than MS15203+Saline thereby showing a reduction in morphine tolerance in female mice. (b) MS15203+Morphine pretreatment was significantly different from MS15203+Saline in male mice, however the magnitude of the shift was reduced (2.33 fold) compared to the Vehicle+Saline and Vehicle+Morphine (five-fold) *n* = 5–8 animals/group.

Following the morphine tolerance induction paradigm, morphine produced a dose-dependent increase in tail-flick latencies in all groups as expected (Fig. 2). There was a statistically significant difference between ED50s in females [*F* (3, 142)  = 6.65, *P*  < 0.001]. A statistically significant difference in ED50s was also found in males [*F* (3, 137) = 29.98, *P* < 0.001]. Mice treated with twice-daily injections of morphine for 4 days showed a rightward shift in the dose-response curve compared to the saline-treated groups for both female (Fig. 2a, Table 1) and male mice (Fig. 2b, Table 1). However, the magnitude of the rightward shift in the dose-response curve between Vehicle+Saline and Vehicle+Morphine was greater in males (five-fold) compared to females (<two-fold shift). Pretreatment with MS15203+Morphine did not produce a statistically significant shift in the morphine dose-response compared to MS15203+Saline in female mice (Fig. 2a; see Table 1 to compare ED50 values). In males, MS15203+Morphine produced a significant shift compared to MS15203+Saline, but it was greatly reduced (2.3-fold) compared to the morphine shift in control animals (five-fold) (Fig. 2b; Table 1).

**Table 1:** Comparison of ED50 values for tail flick test

Treatment	Females	Males
Vehicle+Saline	3.92 ± 0.75 (7)	6.72 ± 1.11 (8)
Vehicle+Morphine	7.16 ± 1.62^a^ (7)	33.73 ± 23.09^a^ (8)
MS15203+Saline	3.34 ± 0.82 (8)	9.45 ± 4.36 (5)
MS15203+Morphine	4.61 ± 1.40^b^ (7)	21.98 ± 7.84^b^ (8)

ED50 values are presented in mg ± 95% CI (sample size).

a *P* < 0.05 from respective saline treated group.

b *P* < 0.05 from Vehicle+Morphine group.

### GPR171 agonist does not alter morphine tolerance on the hot plate test

**Fig. 3:**
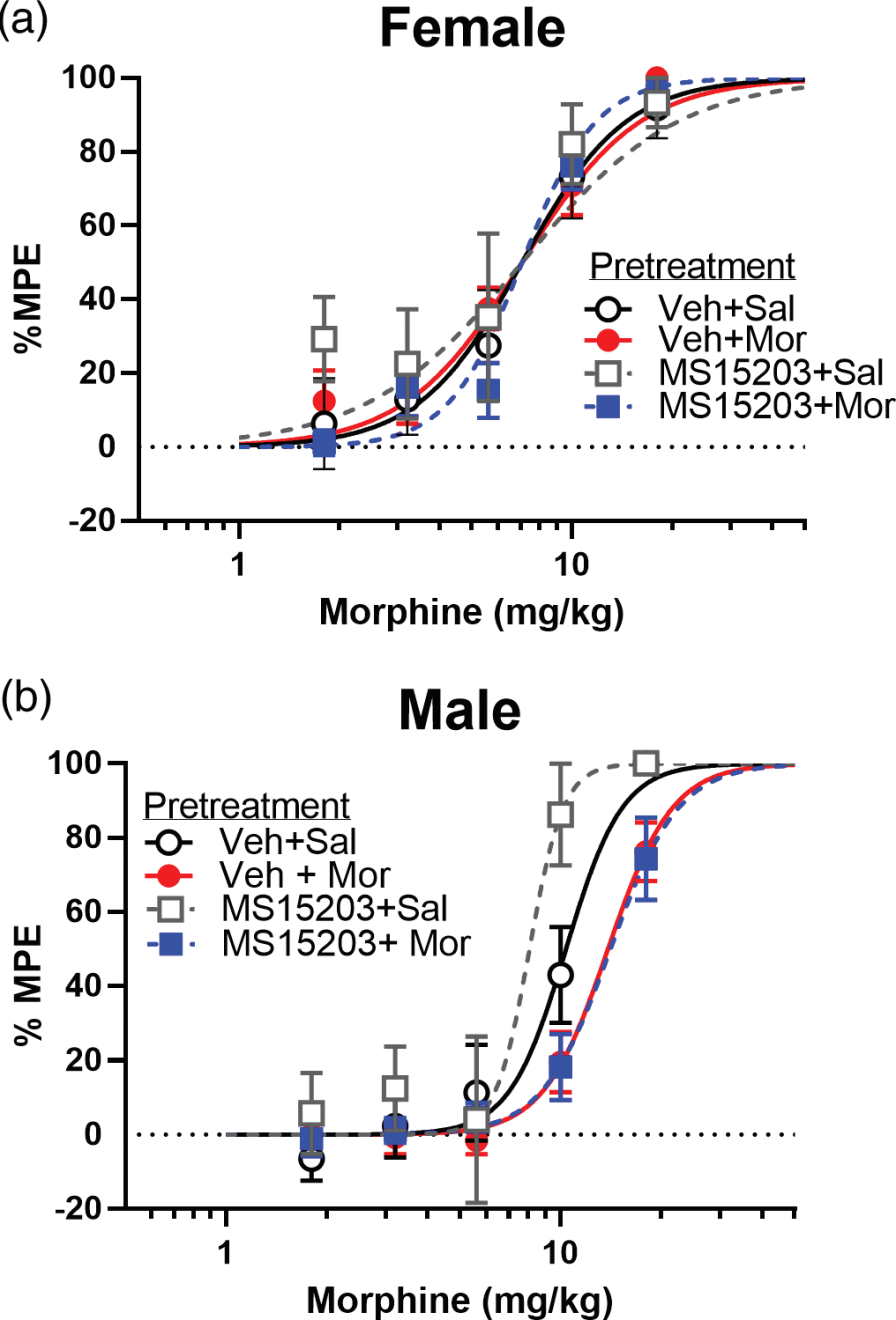
GPR171 agonist, MS15203, had no effect on morphine tolerance on the hot plate test in female or male mice. Morphine tolerance was induced by twice-daily injections of morphine for 4 consecutive days. On day 5, a cumulative quarter-log doses of morphine (1, 1.8, 3.2, 5.6,10 and 18 mg/kg) were injected subcutaneously to perform a dose-response curve. (a) In females, there were no differences between any treatment group indicating a lack of morphine tolerance. (b) Morphine treated male mice exhibited development of tolerance by a rightward shift in the dose response curve compared to the Vehicle+Saline treated group. A similar rightward shift was found between MS15203+Saline and MS5203+Morphine group, suggesting that morphine tolerance is not affected by repeated administration of MS15203 in male (b) mice on the hot plate test. *n* =  5–9 animals/group.

On the hot plate test, there was no statistically significant difference between any repeated drug treatments in female mice, indicating a lack of morphine tolerance on this test [*F* (3, 147) = 1.28, NS] (Fig. 3a; Table 2). There was a significant difference in drug treatments in male mice [*F* (3, 147) = 13.30, *P* < 0.001]. Male mice treated with 4 days of morphine showed a small 1.33-fold rightward shift in the dose-response curve compared to the Vehicle+Saline treated group (Fig. 3b; Table 2). Pretreatment with MS15203+Morphine also produced a significant rightward shift compared to MS15203+Saline (Table 2). Similarly, MS15203+Morphine did not alter ED50 values compared to the Vehicle+Morphine treated group.

**Table 2:** Comparison of ED50 values for hot plate test

Treatment	Females	Males
Vehicle+Saline	7.34 ± 1.85 (7)	10.37 ± 1.49 (9)
Vehicle+Morphine	6.61 ± 1.15 (7)	13.84 ± 1.5^a^(9)
MS15203+Saline	5.74 ± 2.35 (8)	8.07 ± 2.70 (5)
MS15203+Morphine	7.81 ± 0.92 (9)	14.07 ± 1.85^a^(8)

ED50 values are presented in mg ± 95% CI (sample size).

a *P* < 0.05 from respective saline treated group.

### GPR171 agonist does not alter morphine withdrawal in either male or female mice

**Fig. 4:**
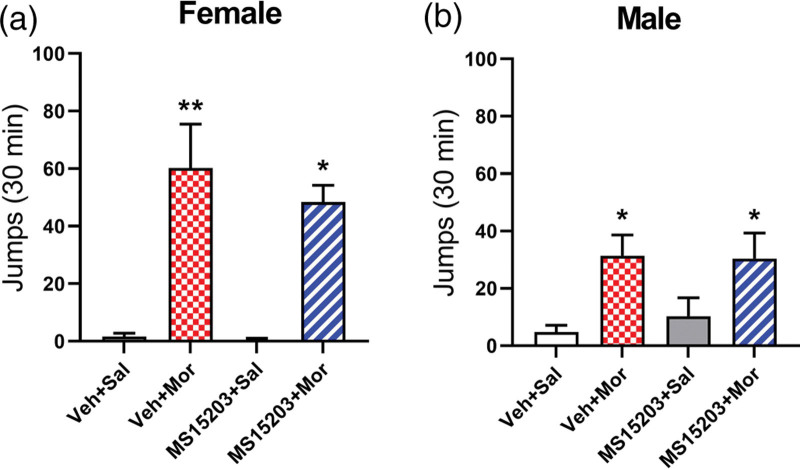
Pretreatment with GPR171 agonist, MS15203, did not alter withdrawal behaviors in either female or male mice. Repeated injection of Vehicle+Morphine caused an increase in the number of jumps compared to the Vehicle+Saline treated group both in female (a) and male (b) mice indicating that those mice developed morphine dependence. Administration of MS15203+Morphine did not alter jumping compared to Vehicle+Morphine treated group in female (a) or male (b) mice. Administration of MS15203+Saline did not increase the number of jumps compared to the Vehicle+Saline treated mice, *P < 0.05, compared with Vehicle+Saline. Data are presented as mean ± SEM. *n* = 5–10 animals/group.

Mice treated with twice-daily injections of Vehicle+Morphine for 4 consecutive days exhibited morphine dependence indicated by a significant increase in the number of jumps compared to the Vehicle+Saline treated female [*F* (3, 25) = 8.71, *P*  < 0.001] and male [*F* (3, 33) = 6.48, *P* < 0.001] mice. Pretreatment with MS15203+Morphine produced an increase in jumping compared to Vehicle+Saline (Tukey’s *P* < 0.05), however, it was not significantly different compared to the Vehicle+Morphine treated group (Tukey’s, NS) in either sex. In addition, repeated administration of MS15203 alone did not cause a significant change in jumping compared to the Vehicle+Saline treated group (Tukey’s, NS). These results indicate that the GPR171 agonist, MS15203, does not alter morphine withdrawal in either female or male mice (Fig. 4a,b).

## Discussion

In this present study, we investigated whether GPR171 modulates morphine antinociception, tolerance and withdrawal in female and male mice. Consistent with our previous findings in male mice (McDermott *et al.*, 2019), we found that a GPR171 agonist, MS15203, enhances morphine antinociception in female mice on the tail-flick test at one time point. Our data also demonstrate that pretreatment with MS15203 attenuates morphine tolerance on the tail-flick test, but not the hot plate test, in female and male mice. However, we found that treatment with the GPR171 agonist does not alter morphine withdrawal in either sex.

Our previous study demonstrated that in male mice a GPR171 agonist, MS15203, enhances morphine antinociception, whereas antagonism of this receptor decreases morphine antinociception during acute morphine treatment on both hot plate and tail-flick tests (McDermott *et al.*, 2019). Here in this study, we evaluated the time course of MS15203 on acute morphine antinociception in female mice and found that the same dose of MS15203 enhances the duration of morphine antinociception on the tail-flick test, but not on the hot plate test. The morphine dose (5 mg/kg) used in this study produced a maximal effect on the tail flick at early time points, therefore making it difficult to evaluate the enhancing effects of MS15203. Overall, the results of these two studies suggest that the GPR171 agonist enhances morphine-induced antinociception in both sexes despite acute administration of this dose of MS15203 (10 mg/kg, i.p.) not having any analgesic properties on its own on these thermal pain tests (McDermott *et al.*, 2019). The diminished antinociceptive effects in females within this study are aligned with our recent study showing that MS15203 alleviated inflammatory and neuropathic pain in male mice, but not female mice (Ram *et al.*, 2021). It is unclear why this effect of MS15203 treatment would be different between sexes or pain tests. However, the pharmacokinetics and pharmacodynamics of MS15203 are unknown, which could contribute to the differences in its effects on the brain versus spinal cord and subsequently differences in hotplate and tail flick tests. It has been shown that MS15203 crosses the blood-brain barrier and activates neurons in the hypothalamus (Wardman *et al.*, 2016). Our hypothesis is that administration of MS15203 leads to enhanced downstream signaling which in turn causes enhanced morphine antinociception.

The main goal of this study was to evaluate the long-term effects of coadministration of MS15203 and morphine. To remain consistent, we used the same doses of both drugs that showed enhancement of morphine antinociception. Repeated injections over 4 days produced a significant difference in ED50 values between saline versus morphine on the tail-flick test in both sexes. Our data show that repeated activation of GPR171 with the agonist, MS15203, attenuated this morphine-induced tolerance in female and male mice on the tail-flick test. In females, there is no difference between the ED50s for MS15203+Saline and MS15203+Morphine indicating a lack of the development of tolerance in the presence of MS15203. Whereas in males there is still a significant difference between these groups although the shift is greatly reduced from a five-fold to a two-fold shift. This reduction in the shift of the dose-response curve could be interpreted as a decrease in tolerance or an increase in antinociception. Given that MS15203 increases morphine’s pain-relieving property during acute treatment, MS15203 could lead to a corresponding progressive increase in morphine antinociception during repeated administration of MS15203+Morphine, resulting in enhanced morphine antinociception on day 5.

Our previous immunohistochemistry data show that GPR171 is expressed on the neurons containing Gamma-Aminobutyric Acid (GABA) of PAG (McDermott *et al.*, 2019). It is evident that mu-opioid receptors (MOPr) are also located on the GABAergic neurons in the PAG (Stiller *et al.*, 1996; Lueptow *et al.*, 2018). Due to the co-expression of these two receptors in the GABAergic neurons of the PAG, activation of these two receptors together may inhibit GABA release to a greater extent than morphine alone. Thereby, increasing morphine antinociception during acute treatment and in parallel increasing morphine antinociception during chronic treatment. Our results show that repeated MS15203 does not alter morphine ED50 values in male mice on the hot plate test. These test-dependent differences may be caused by differences in GPR171 expression across the nervous system which remains to be characterized. Within the spinal cord of male mice, GPR171 is expressed in different populations of cells that express various transient receptor potential (TRP) channels and can reduce nociception mediated by these channels (Cho *et al.*, 2021). This suggests that GPR171 is acting differently in the brain and spinal cord which could be the mechanism by which we found differences in the hotplate and tail-flick tests. Taken together, these results indicate that combined and repeated treatment with these doses of MS15203 and morphine does not enhance morphine tolerance. Further, this combination therapy has the potential to reduce the dose of morphine needed and attenuate tolerance after long-term treatment.

Interestingly in this study, we found that overall female mice developed less morphine tolerance compared to male mice regardless of GPR171 agonist treatment. Previous literature on sex differences in morphine tolerance are controversial and have reported mixed findings. A number of studies have shown that chronic administration of morphine induces greater and faster tolerance in male rats than in female rats (Badillo-martinez *et al.*, 1984; Craft *et al.*, 1999; South *et al.*, 2001; Loyd *et al.*, 2008), whereas other studies show no sex difference in tolerance (Thornton and Smith, 1997; Holtman *et al.*, 2004). The greater sensitivity of males to acute morphine antinociception could account for the greater tolerance observed in males. Although we did not examine the estrous cycle phase of the female mice in this current experiment, it should be noted that female rats differ in the degree of morphine tolerance depending on the phase of the estrous cycle (Shekunova and Bespalov, 2004). One possible mechanism for the dissimilar tolerance development observed in female mice on the tail flick and hot plate test may be explained by the supraspinal versus spinal nature of the two tests. The hot plate test is a supraspinal pain assay whereas tail-flick measures a spinal reflex. These two methods predominantly reflect nociception and tolerance at different levels of the central nervous system (Chapman *et al.*, 1985; Le Bars *et al.*, 2001). On the hot plate test, male mice treated with morphine showed ~two-fold shift in the ED50 values, whereas female mice showed no difference compared to saline-treated mice (Table 2). On the tail-flick test, male and female mice showed a five-fold and less than a two-fold shift in the ED50 values, respectively (Table 1). Considering all these above-mentioned factors, it can be said that lack of tolerance in females might be a result of many associated factors.

Our withdrawal experiment shows that MS15203 does not alter morphine withdrawal-induced jumping in either male or female mice. Although the PAG is thought to perform a key role in morphine withdrawal (Ouyang *et al.*, 2012), other studies have highlighted the role of locus coeruleus, ventral tegmental area, amygdala, frontal cortex and the spinal cord (Ammon *et al.*, 2003; McClung *et al.*, 2005; McPhie and Barr, 2009). By immunohistological analysis, we have only explored the expression of GPR171 within the PAG, while the expression of this receptor in other areas is still unknown. A likely explanation to our observation is that morphine withdrawal is mediated largely by the areas other than PAG and therefore MS15203 has minimal influence on this phenomenon due to lower GPR171 expression in those brain areas. Additionally, it has been reported that glutamatergic neurons are involved in naloxone-precipitated withdrawal symptoms (Zhang *et al.*, 2020). Our immunohistochemistry data shows that the GPR171 is primarily found in GABAergic neurons and, to a lesser extent, in glutamatergic neurons of PAG (McDermott *et al.*, 2019) therefore activation of this receptor might have minimal effect on morphine withdrawal. It is evident that morphine inhibits adenylyl cyclase activity and cAMP production following acute treatment (Fedynyshyn and Lee, 1989), but there is a compensatory increase in adenylyl cyclase signaling following chronic treatment of morphine causing an upregulation of cAMP during withdrawal (Terwilliger *et al.*, 1991). Because GPR171 is also an inhibitory GPCR, that inhibits cAMP production after acute treatment (Gomes *et al.*, 2013), it is possible that the reduction in the adenylyl cyclase signaling by the GPR171 agonist is not potent enough to attenuate cAMP after morphine withdrawal. Overall, these data suggest that this dose of MS15203 can be safely used as a combination therapy with morphine without worsening morphine withdrawal.

The cumulative results obtained here support the idea that GPR171 and MOPr receptor systems work together. Our previous *in vitro* study reported that antagonism or knockdown of GPR171 reduces MOPr-mediated G protein signaling (McDermott *et al.*, 2019), indicating that GPR171 may be a regulator of MOPr signaling. This regulation could result from the two receptors interacting in a heterodimer formation. Both MOPr and GPR171 have been found to functionally interact with other receptors and form heterodimers (Fujita *et al.*, 2015; Gomes *et al.*, 2016; Margolis *et al.*, 2017). These receptor–receptor interactions modulate ligand binding and the signaling properties of the individual receptors (Al-Hasani and Bruchas, 2011; Stockton and Devi, 2012). Further, it has been previously reported that MOPrs can be modulated by other receptors which can alter antinociception and tolerance such as delta-opioid receptors (Porreca *et al.*, 1984; Abul-Husn *et al.*, 2007), cannabinoid receptors (Cichewicz and McCarthy, 2003), glutamate receptors (Nishiyama *et al.*, 1998; Morgan *et al.*, 2009), orexin receptors (Azhdari-Zarmehri *et al.*, 2013; Emam *et al.*, 2016) and alpha-2 adrenergic receptors (Drasner and Fields, 1988). We know that GPR171 can interact with another GPCR, GPR83 (Gomes *et al.*, 2016), but it is unknown whether it dimerizes with the MOPr as well. Because they both are found within GABA neurons in the PAG neurons to regulate antinociception it is possible that they directly interact (Morgan *et al.*, 2008; Bobeck *et al.*, 2014; McDermott *et al.*, 2019). Activation of these two receptors together would likely facilitate crosstalk between signaling pathways and inhibit GABA release to a greater extent thereby exciting the projection neurons from the PAG to the rostral ventromedial medulla leading to enhanced antinociception (Terwilliger *et al.*, 1991; Lau and Vaughan, 2014; Lueptow *et al.*, 2018).

Several alternative explanations for these results must be addressed. In this experiment, we showed that repeated pretreatment with MS15203+Morphine decreases morphine ED50 values compared to morphine alone, which could be interpreted as a decrease in tolerance. However, because we do not know the pharmacokinetics and the resulting half-life of MS15203, other explanations are available. In the current study and previously, we showed that MS15203 increases morphine antinociception (McDermott *et al.*, 2019). Therefore, if MS15203 remains bioavailable for more than 24 h its presence may, rather than decreasing morphine tolerance, be responsible for the sustained enhancement of morphine antinociception. While MS15203 selectivity for the GPR171 has been verified *in vitro* and shows a low binding affinity for the MOPr (Wardman *et al.*, 2016), this selectivity of MS15203 has not been validated *in vivo*. In addition, it is unknown whether morphine and MS15203 are producing their effects within the same or different neurons or even glia. Recent evidence shows morphine tolerance coincides with glial activation and inflammation that undermine the antinociceptive effects of morphine (Eidson and Murphy, 2013; Averitt *et al.*, 2019).

Several limitations of this study require future exploration. For example, we used only one dose, 10 mg/kg, of MS15203 and dose administration timeline due to the known efficacy of this dose at increasing morphine antinociception and decreasing chronic pain (McDermott *et al.*, 2019; Ram *et al.*, 2021). However, whether 10 mg/kg of MS15203 over 5 days is optimal for altering morphine tolerance or withdrawal is unknown. Future studies should use varying dosages of both drugs and alterations in the timing of administration and testing to further investigate the role of GPR171 in morphine tolerance. The cutoff values for the hotplate and tail-flick tests were set arbitrarily to prevent tissue damage by sustained exposure. Therefore the calculation of ED50 values was based on those cutoff latencies to compare across different drug conditions, as done previously (Bobeck *et al.*, 2014). However, this may have lead to an inaccurate ED50 since some groups (i.e. male morphine on tail-flick) did not reach the cutoff at the highest morphine dose. Using the cumulative dosing procedure it was not possible to add additional morphine doses to the dose response because all groups were injected with the exact same doses for both tests.

Another limitation is that only thermal pain tests were used, which may result in different results than mechanical or chronic pain tests (see Ram *et al*, 2021). Lastly, as was mentioned above, pretreatment with MS15203+Morphine could be due to the lingering effects of MS15203. Pharmacokinetic studies should be conducted to understand how MS15203 is metabolized and absorbed throughout the brain and body.

In conclusion, a GPR171 agonist, MS15203, decreases morphine tolerance in female and male mice on the tail-flick test, and, notably, it does not enhance morphine-induced tolerance or withdrawal during long-term treatment. Future studies are needed to assess different dose combinations of these drugs to determine if this combination therapy could be used to reduce opioid dosage, which would in turn allow smaller doses of opioid agonists to produce the same amount of antinociception.

## Acknowledgements

This research was funded by a Pharmacology and Toxicology research startup grant from the PhRMA Foundation, NIH National Center for Advancing Translational Sciences (TR003667) and from startup funds from the Utah State University.

### Conflicts of interest

There are no conflicts of interest.
